# Imbalance of proresolving lipid mediators in persistent allodynia dissociated from signs of clinical arthritis

**DOI:** 10.1097/j.pain.0000000000001908

**Published:** 2020-05-04

**Authors:** Benjamin L. Allen, Karli Montague-Cardoso, Raffaele Simeoli, Romain A. Colas, Silvia Oggero, Bruno Vilar, Peter A. McNaughton, Jesmond Dalli, Mauro Perretti, Emanuele Sher, Marzia Malcangio

**Affiliations:** a Wolfson Centre for Age Related Diseases, King's College London, London, United Kingdom; b Laboratory of Metabolic Biochemistry Unit, Department of Pediatric Medicine, Bambino Gesù Children's Hospital, IRCCS, Rome, Italy; c Barts and The London School of Medicine, The William Harvey Research Institute, Queen Mary University of London, London, United Kingdom; d Neuroscience Discovery, Lilly Research Centre, Eli Lilly and Company Ltd, Surrey, United Kingdom

**Keywords:** Macrophages, Arthritis pain, Inflammation, Resolution, Lipid mediators

## Abstract

Rheumatoid arthritis-associated pain is poorly managed, often persisting when joint inflammation is pharmacologically controlled. Comparably, in the mouse K/BxN serum-transfer model of inflammatory arthritis, hind paw nociceptive hypersensitivity occurs with ankle joint swelling (5 days after immunisation) persisting after swelling has resolved (25 days after immunisation). In this study, lipid mediator (LM) profiling of lumbar dorsal root ganglia (DRG), the site of sensory neuron cell bodies innervating the ankle joints, 5 days and 25 days after serum transfer demonstrated a shift in specialised proresolving LM profiles. Persistent nociception without joint swelling was associated with low concentrations of the specialised proresolving LM Maresin 1 (MaR1) and high macrophage numbers in DRG. MaR1 application to cultured DRG neurons inhibited both capsaicin-induced increase of intracellular calcium ions and release of calcitonin gene-related peptide in a dose-dependent manner. Furthermore, in peritoneal macrophages challenged with lipopolysaccharide, MaR1 reduced proinflammatory cytokine expression. Systemic MaR1 administration caused sustained reversal of nociceptive hypersensitivity and reduced inflammatory macrophage numbers in DRG. Unlike gabapentin, which was used as positive control, systemic MaR1 did not display acute antihyperalgesic action. Therefore, these data suggest that MaR1 effects observed after K/BxN serum transfer relate to modulation of macrophage recruitment, more likely than to direct actions on sensory neurons. Our study highlights that, in DRG, aberrant proresolution mechanisms play a key role in arthritis joint pain dissociated from joint swelling, opening novel approaches for rheumatoid arthritis pain treatment.

## Abstract 2

Supplemental Digital Content is Available in the Text.

In inflammatory arthritis, we show an imbalance of proresolving bioactive lipid mediators in dorsal root ganglia, under persistent nociceptive states, and antinociceptive effects of maresin-1 administration.

## 1. Introduction

Rheumatoid arthritis (RA) is a chronic, autoimmune disease with pain representing a persistent and debilitating symptom, which significantly impacts the quality of life of patients; it is common for many patients to avoid tasks so as to limit any activity-induced pain. Patients often report pain even when overt joint inflammation is in remission after biologic or nonbiologic disease-modifying antirheumatic drug treatment.^15,29^ While the intense pain which is associated with flares of the disease can be controlled with glucocorticoids and analgesics, less controlled is the constant experience of pain with its daily manifestation of dull ache. During acute synovitis pain intensity is strongly associated with inflammation severity, but before and after inflammatory disease suppression, the association between pain and joint inflammation is often only weak.^29^ Existing therapies for pain in RA consist largely of non-steroidal anti-inflammatory drugs (NSAIDs), which besides having undesirable side-effects, also show limited efficacy in alleviating chronic pain, partly because of their discontinuation, or reduced dosage after the flare phases terminate.^27^ At present, alternative therapies to NSAIDs include tumour necrosis factor (TNF) blockers^5^; however, this treatment also increases the risk of serious side-effects (eg, upper respiratory tract infection).^1^ Novel therapies to treat pain that persists in RA, when joint inflammation is controlled, are therefore urgently needed.

In RA, pain is initially driven by synovial inflammation and sensitisation of sensory neurons innervating the joint, whose cell bodies are located in the dorsal root ganglia (DRG) (peripheral sensitisation) and then maintained by concomitant neuronal plasticity at the first sensory synapse within the spinal cord (central sensitisation).^16,18^ Preclinical evidence indicates that neuroimmune interactions contribute significantly to both peripheral and central sensitisation. Specifically, monocytes/macrophages infiltrated in sensory neurons contribute to peripheral sensitisation by the release of proinflammatory mediators such as cytokines.^13,22^ In addition, in rodent arthritic joints, proinflammatory bioactive lipid mediators (LMs) contribute to heightened states of inflammation.^19^ Clinically, bioactive LMs are found in the synovial fluid of patients with RA.^19^ Despite this accumulated knowledge, our understanding of the mechanisms underlying RA pain, particularly the pain that persists when overt inflammation has subsided, is still incomplete.

In this study, with the aim to delineate mechanisms underlying pain dissociated from inflammation, we assessed bioactive LM profiles in DRG using the mouse K/BxN serum-transfer model of inflammatory RA that features transient arthritic swelling in the joints and concomitant hind paw mechanical allodynia, but also significant allodynia, which persists after resolution of joint swelling.^4^

## 2. Methods

### 2.1. Animals

All experiments were performed on adult male C57BL/6 mice (Envigo, Cambridgeshire, United Kingdom) (10-12 weeks old, approximately 25 g). Mice were housed in groups of up to 5 per standard cage. All animals were kept at room temperature with a 12-hour light/dark cycle. Animals received food and water ad libitum.

### 2.2. Induction of K/BxN serum-transfer model of inflammatory arthritis

K/BxN serum was obtained as previously described.^19^ Serum-transfer–induced arthritis was performed, in naïve mice, by 50-µL intraperitoneal injection of arthritogenic serum on days 0 and 2. Control mice were given equal volume injections containing pooled sera from KRN/C57 mice. Clinical signs of arthritis were evaluated using a 12-point scoring system.^19^ Each limb is scored separately; 0 to 3 points per limb with the following criteria: 0—no sign of redness/swelling; 1—redness/swelling observed in either ankle/wrist, pad, or any of the digits; 2—redness/swelling in 2 regions; and 3—redness/swelling seen in all limb regions. Scores for all 4 limbs were combined to give a total score, maximum 12 per animal.

### 2.3. Intraperitoneal administration of maresin 1

After K/BxN serum transfer, mice were treated intraperitoneally with 100 ng MaR1 (Cayman Chemical, Ann Arbor, MI) in saline. K/BxN controls were administered with saline (intraperitoneally). For experiments using protocol 1, mice received MaR1 treatment on days 5, 7, 9, and 11 after K/BxN serum injection. Whereas, using Protocol 2, MaR1 treatment occurred on days 19, 21, and 23. On each treatment day, MaR1 was freshly prepared from stock solution in ethanol and stored under nitrogen at −80°C without exposure to light. All behavioural testing was performed 1 hour after MaR1 injection.

### 2.4. Oral administration of gabapentin

On days 19, 20, and 21 after K/BxN serum injection, mice were treated orally with gabapentin (60 mg/kg orally; LKT Laboratories, St Paul, MN) dissolved in distilled water. Vehicle groups received distilled water (orally). Baseline paw withdrawal thresholds were taken on day 18 after K/BxN serum transfer, where clinical signs of arthritis (overt swelling/redness) had subsided. On each treatment day, behavioural testing was performed 1 hour after oral administration. Behavioural testing was also performed 24 hours after the final gabapentin treatment, on day 22.

### 2.5. Behavioural testing

Hind paw mechanical withdrawal thresholds were assessed by applying a series of calibrated von Frey filaments (0.02-1.0 g; North Coast Medical Morgan Hill, CA) to the plantar surface of the hind paw. On each day of testing, animals were habituated for 30 minutes, before application of initial 0.07-g filament. Filaments were then applied at increasing intensity until a withdrawal response was achieved or application of 1.0-g filament failed to elicit a withdrawal response. Fifty percent paw withdrawal threshold was calculated using the Dixon up and down method.^2^ Briefly, if a withdrawal response to a stimulus was established, the paw was retested with the next lowest intensity filament below stimuli that elicited a withdrawal, until no withdrawal occurred. At which, point stimuli with ascending force filaments were applied until a response was observed. Fifty percent withdrawal threshold calculation by the Dixon method requires a minimum of 5, upto a maximum of 9 filament applications. The experimenter was blinded to treatment groups.

### 2.6. Immunohistochemistry

For immunohistochemistry in perfuse-fixed tissue, mice were transcardially perfused with saline solution followed by 4% paraformaldehyde (VWR Chemicals, Leicestershire, United Kingdom) with 1.5% picric acid in 0.1 M phosphate-buffered saline (PBS) before L3, L4, and L5 DRGs were excised. Transverse DRG sections (10 µm) were then cut using a cryostat (Bright Instruments, Luton, United Kingdom) and mounted onto Superfrost Plus microscope slides (Thermo-Scientific, Leicestershire, United Kingdom). Sections were then blocked with 1% bovine serum albumin (BSA) (Sigma-Aldrich, Dorset, United Kingdom) for 1 hour then incubated overnight with rat anti-mouse F4/80 (1:400; Abcam, Cambridge, United Kingdom), followed by anti-rat Alexa Flour 488 secondary antibody (1:1000; Invitrogen, Paisley, United Kingdom). Slides are then washed 3 times for 10 minutes in 0.1% PBS-T followed by incubation for 2 hours at room temperature in the appropriate secondary antibodies (Alexa-Flour 546 conjugated [Life Technologies, Paisley, United Kingdom]). All antibodies were prepared in 0.1 M PBS with 0.1% BSA and 0.1% Triton X-100 (Sigma). For negative controls, the primary antibody was omitted; this resulted in the absence of staining. Images for immunofluorescence analysis were captured using a Zeiss Axioplan 2 fluorescence microscope and analysed using ImageJ software (1.50i, Wayne Rasband, National Institutes of Health, USA). F4/80+ profiles, indicative of number of macrophage cells, were quantified within fixed areas (4 × 10^4^ µm) per section. At least 4 sections from 3 mice per group were analysed.

### 2.7. Targeted liquid chromatography mass spectrometry–based lipidomics of lumbar dorsal root ganglia

On day 5 and 25 after K/BxN induction, L4 and L5 DRG were dissected and immediately frozen in liquid nitrogen. All samples for liquid chromatography mass-spectrometry (LC-MS/MS)–based metabololipidomics were processed as previously described.^7^ Before sample extraction, deuterated internal standards, representing each region in the chromatographic analysis (d4-LTB_4_, d8-5S-HETE, d4-PGE_2_, d5-LXA_4_, d5-RvD2, d5-LTC_4_, d5-LTD_4_, and d5-LTE_4_, 500 pg each), were added to facilitate quantification in cold methanol. Samples were gently homogenised using a glace dounce and kept at −20°C for a minimum of 45 minutes to allow for protein precipitation. Samples were then centrifuged for 10 minutes at 4000×*g*. Supernatants were subjected to solid-phase extraction, methyl formate and methanol fractions collected, brought to dryness, and resuspended in phase (methanol/water, 1:1, vol/vol) for injection on a Shimadzu LC-20AD high performance liquid chromatography (HPLC) and a Shimadzu SIL-20AC autoinjector, paired with a QTrap 6500 (ABSciex, Warrington, United Kingdom). For the methyl formate LMs, an Agilent Poroshell 120 EC-C18 column (100 × 4.6 mm × 2.7 μm) was kept at 50°C, and LMs were eluted with a mobile phase consisting of methanol-water-acetic acid of 50:50:0.01 (vol/vol/vol) that was ramped to 80:20:0.01 (vol/vol/vol) from 2 to 11 minutes, maintained till 14.5 minutes, and then rapidly ramped to 98:2:0.01 (vol/vol/vol) for the next 0.1 minutes. This was subsequently maintained at 98:2:0.01 (vol/vol/vol) for 5.4 minutes, and the flow rate was maintained at 0.5 mL/minute. QTrap 6500 was operated in negative ionisation mode using a multiple reaction monitoring method as previously described.^7^ For the methanol LMs, an Agilent Poroshell 120 EC-C18 column (100 × 4.6 mm × 2.7 μm) was kept at 50°C, and conjugates were eluted with a mobile phase consisting of methanol-water-acetic acid of 55:45:0.5 (vol/vol/vol) that was isocratic for 1 minute, ramped to 70:30:0.1 (vol/vol/vol) over 5 minutes, then to 80:20:0.5 (vol/vol/vol) for 2 minutes, then isocratic 80:20:0.5 (vol/vol/vol) for the next 3 minutes, and ramped to 98:2:0.5 (vol/vol/vol) over 3 minutes. This was subsequently maintained at 98:2:0.5 (vol/vol/vol) for 3 minutes. The flow rate was maintained at 0.6 mL/minute. QTrap 6500 was operated in positive ionisation mode using a multiple reaction monitoring method as previously described^6^ Each LM was identified using strict criteria including matching retention time to synthetic and authentic materials and at least 6 diagnostic ions. Calibration curves were obtained for each using synthetic compound mixtures and deuterium-labeled LM at 0.78, 1.56, 3.12, 6.25, 12.5, 25, 50, 100, and 200 pg. Linear calibration curves were obtained for each LM, which gave r2 values of 0.98 to 0.99.

### 2.8. Preparation of dorsal root ganglia neuronal cell cultures

From naïve mice, all DRG were dissected aseptically and placed in Hibernate A (Gibco; Thermo-Fisher) supplemented with 0.5 mM L-Glutamine (Gibco; Thermo-Fisher) and 2% B-27 Supplement (50X) (Gibco; Thermo-Fisher). Dorsal root ganglia were dissociated in 2-mg/mL papain in Hank's balanced salt solution (HBSS) (30 minutes; 37°C & 5% CO_2_) followed by 2.5-mg/mL collagenase in HBSS (30 minutes; 37°C & 5% CO_2_). Cells were triturated and then resuspended in Neurobasal A (Gibco; Thermo-Fisher) supplemented with 0.5 mM L-Glutamine (Gibco; Thermo-Fisher) and 2% B-27 Supplement (50X) (Gibco; Thermo-Fisher). Cell suspensions were then seeded onto laminin-coated glass coverslips and incubated for 30 minutes (37°C & 5% CO_2_) before overnight incubation in Neurobasal medium. For experiments involving pertussis toxin (PTX) before incubation, before calcium imaging, cells were incubated with 500-ng/mL PTX (EMD Millipore, Burlington, MA) in Neurobasal medium for 18 hours at 37°C, with controls incubated in Neurobasal medium plus sterile H_2_O vehicle.

### 2.9. Calcitonin gene-related peptide release

Following 48 hours in culture, medium was replaced with fresh medium for 10 minutes, 3 times, to obtain 3 basal fractions. Cultures were then stimulated for 10 minutes with capsaicin (1 μM) in the absence or presence of MaR1 (1, 10, and 100 nM), gabapentin (1 and 10 nM), or vehicle. The medium was then collected (release fraction) and replaced with fresh medium twice, for 10 minutes each time (recovery fractions). The calcitonin gene-related peptide (CGRP) content in each fraction was then measured using an enzyme-linked immunosorbent assay (Reddot Biotech, Inc., British Columbia, Canada catalogue No: RD-CGRP-Mu) according to manufacturer's instructions. The experiment was repeated in triplicate with each sample being run in duplicate. Calcitonin gene-related peptide levels were expressed as CGRP increase relative to mean basal fraction for each sample.

### 2.10. Calcium imaging

On the experimental day, DRG neurons were loaded with fura-2 AM dye (3 µM, 30 minutes) in the presence of pluronic acid F-127 0.02% (Invitrogen) at 37°C and 5% CO_2_. Coverslips were imaged using Nikon Eclipse Ti-E inverted microscope at 10x magnification and analysed using ImageJ using ImageJ software (1.50i, Wayne Rasband, National Institutes of Health, USA). While imaging, cells were continuously superfused with either HBSS, 500 nM capsaicin, 50 mM KCl, or 0.3- to 3-ng/mL MaR1 at a rate of 2 mL/minute. Capsaicin responses were quantified as the difference (Δ[− [Ca^2+^]]i) between baseline, calculated as F340/380 ratio immediately before application of capsaicin and peak calcium after capsaicin application. Capsaicin (500 nM) was applied 6 times for 15 seconds at 5-minute intervals. After sixth capsaicin application, cells are exposed to 50 mM KCl to confirm cell viability. In MaR1 experiments, coverslips are continuously superfused with 0.3- to 3-ng/mL MaR1 in HBSS for 1 minute before, and 1 minute after, fourth capsaicin application. For each coverslip, MaR1 was freshly prepared from stock solution in ethanol stored under nitrogen at −80°C without exposure to light.

### 2.11. Primary culture of peritoneal macrophages

Macrophages were collected from mice through lavage of the peritoneal cavity with 1% penicillin/streptomycin sterile saline. Macrophages were seeded in 12-well plates at 1 × 10^6^ cells/well and allowed to adhere overnight. Then, nonadherent cells were removed by washing and adherent macrophages covered with macrophage medium, consisting of red phenol-free complete Dulbecco's modified eagle's medium (Gibco) supplemented with 10% heat-inactivated fetal bovine serum (Gibco; Thermo-Fisher), 1% pen/strep (Gibco; Thermo-Fisher), and 1% sodium pyruvate (Gibco; Thermo-Fisher). Cells were then incubated for 3 hours with 100-ng/mL lipopolysaccharide from *Escherichia coli* (O111:B4; Sigma-Aldrich) (lipopolysaccharide [LPS]). Thereafter, either 3-ng/mL MaR1 or macrophage medium vehicle was added to cells for an additional 5 hours. Culture media were then removed, with cell lysates obtained using a lysis/binding solution provided by mirVana miRNA Isolation Kit (Invitrogen). Total and small RNA were isolated. mRNA and miRNA expression levels were then detected by quantitative polymerase chain reaction.

### 2.12. Real-time quantitative polymerase chain reaction

Real-time PCR experiments were performed as previously described.^26^ Total and small RNA-enriched fractions were isolated using mirVana miRNA Isolation Kit (Invitrogen) and RNA-eluted using RNase-free water. Purity and concentration of RNA samples were estimated using Nano-Drop ND-100 Spectrophotometer (ThermoFisher Scientific). For detection of miR-155, each small RNA template sample was diluted to 5 ng/μL using nuclease-free water, and cDNA synthesised using the miRCURY LNA Universal cDNA Synthesis kit II (Exiqon, Manchester, United Kingdom). PCR for miR-155 was performed using ExiLENT SYBR Green master mix (Exiqon) in a LightCycler 480 (Roche, Welwyn Garden City, United Kingdom). Primers for miR-155 were provided by Exiqon (mmu-miR-155-5p; MIMAT0000165). Duplicates CTs were averaged, and the relative quantities of miRNA were calculated using the 2^−ΔΔCT^ method and normalised to several artificial spiked-in controls. For quantification of mRNA levels, PCR was performed using a LightCycler FastStart DNA MasterPlus SYBR Green I kit (Roche) in a LightCycler 480 (Roche). All primer sequences are reported in Supplemental Table 1 (available at http://links.lww.com/PAIN/B11). Duplicate CTs were averaged, and the results analysed by the 2^−ΔΔCT^ method, normalised to expression within control groups and using Actb as a housekeeper gene.

### 2.13. Flow cytometry for dorsal root ganglia tissue

On day 25 after K/BxN induction, from each mouse, C6-8 and L3-5 DRG were dissected bilaterally, in accordance with the sensory innervation of rodent forepaws and hind paws respectively. Dorsal root ganglia tissue were then dissociated using 3-mg/mL dispase (Roche), 0.1% collagenase (Sigma-Aldrich) and 200-U/mL DNAse I (Roche) in F-12 medium (Life Technologies) for 45 minutes. Dorsal root ganglia were triturated and then centrifuged for 5 minutes at 800 rpm. Cell pellets were then resuspended in 500-µL PBS (no calcium chloride or magnesium chloride) (Sigma) plus 1% BSA. A 30-µL aliquot of cell suspension was taken for cell counting to establish absolute cell numbers for each sample. Remaining cell suspensions were then incubated on ice for 20 minutes with anti-mouse CD16/CD32 (Clone 2.4G2; BD Biosciences, San Jose, CA) to block Fc receptors. Cells were then incubation with a mix of the following fluorochrome-conjugated anti-mouse antibodies for 30 minutes: CD45.1-Pacific Blue (Clone 30-F11; BioLegend, London, United Kingdom), F4/80-PE (Clone BM8; eBioscience), CD11b-APC (Clone M1/70; eBioscience), CD206-PE-Cy7 (Clone C068C2; BioLegend), and CD11c-APC eFluor780 (Clone N418; eBioscience), all used at 1:200 concentration. Cells were then washed, centrifuged (1 minute, 800 rpm), and resuspended in flow buffer before being analysed. Samples were run using LSRFortessa cell analyser (BD Bioscience) and analysed using FlowJo software (v10.1; BD Bioscience).

### 2.14. Flow cytometry for paw tissue

Leukocytes were isolated from arthritic paws after tissue digestion. Paws were cut 3 mm above the heel, skin from the feet was removed, and fingers disarticulated by pulling with blunt forceps. The so prepared foot was incubated in digestion buffer (Collagenase D [Roche; 0.5-μg/mL] and DNAse [Sigma-Aldrich; 40-μg/mL] in serum-free RPMI) with gentle agitation for 60 minutes at 37°C. Cells released during the digestion were filtered through 70-μm cell strainer, centrifuged at 400×*g* for 10 minutes, and resuspended in PBS for counting; dead cells were discriminated using trypan blue. Leukocytes were first stained with Zombie NIR (BioLegend; 1:500, 20 minutes, 4°C) to identify live cells, and distinct leukocyte subtypes were identified using the following antibodies from eBioscience: CD45 PerCP (1-μg/mL, clone 30-F11), CD11b Pe-Cy7 (0.2-μg/mL, clone M1/70), Ly6C eFluor450 (2-μg/mL, clone HK1.4), CD115 BV711 (2-μg/mL, clone AF598), MHCII AF700 (1.25-μg/mL, clone M5/114), F4/80 BV650 (2-μg/mL, clone BM8) and from BD Horizon: Ly6G FITC (5-μg/mL, clone 1A8), CD43 BV510 (2-μg/mL, clone 57), SinglecF PE-CF594 (2-μg/mL, clone E50-2440). Samples were run using LSRFortessa cell analyser (BD Bioscience) and analysed using FlowJo software (v10.1; Tree Start, Inc).

### 2.15. Statistics

All data are presented as mean ± SEM, where n is the number of biological replicates (individual mice), except for experiments using peritoneal macrophages whereby n is the number of experimental replicates, where each experiment uses cells pooled from 5 mice. Statistical analyses were performed with GraphPad Prism (v8.0.0; GraphPad Software) using unpaired Student *t*-test (2 groups using different samples), one-way analysis of variance followed by post hoc Tukey test (more than 2 groups) and two-way repeated-measures analysis of variance followed by post hoc Tukey test for behavioural testing and clinical scoring. Differences between means was considered to reach statistical significance when *P* < 0.05. Orthogonal partial least square to latent structures discriminant analysis was performed using SIMCA 15.0.2 software (Umetrics, Umea, Sweden) after mean centering and unit variance scaling of LM levels.

### 2.16. Study approval

All studies were performed with appropriate ethical approval. Animal studies were performed in accordance with the UK Animals (Scientific Procedures) Act 1986 and local care and use guidelines. Approval for these studies was provided by King's Animal Welfare and Ethical Review Body, London, United Kingdom.

## 3. Results

**Figure 1.**
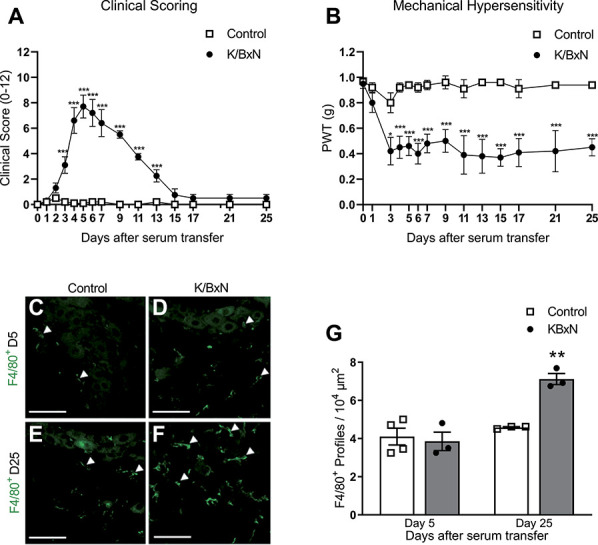
K/BxN serum-transfer arthritis is associated with mechanical hypersensitivity and macrophage joint swelling in DRG when joint swelling is resolving in the hind paw. (A) Clinical signs of arthritis after K/BxN serum transfer (2 × 50-µL intraperitoneal injections on days 0 and 2) evaluated using a 12-point clinical arthritis scoring of mouse paws (B) Mechanical hypersensitivity assessed using von Frey filaments after K/BxN serum transfer. *P < 0.05 or ***P < 0.001 vs same day control, two-way repeated-measures ANOVA, post hoc Tukey. Data are expressed as mean ± SEM; n = 10 male mice per group. Representative images of F4/80^+^ profiles (arrows) (macrophages) infiltrated in DRG on (C and D) day 5 and (E and F) day 25 after serum transfer. Scale bars, 50 μm. (G) Quantification of macrophages in lumbar DRG at 5 and 25 days after K/BxN serum transfer. *P < 0.05 vs same day control, one-way ANOVA, post hoc Tukey. Data are expressed as mean ± SEM; n = 4 mice per group. ANOVA, analysis of variance; DRG, dorsal root ganglia.

Systemic administration of serum obtained from K/BxN mice was associated with joint swelling and mechanical hypersensitivity. Specifically, mice exhibited significant fore and hind paw joint swelling as early as from 24 hours after serum transfer, with a peak at day 5, and slowly subsided thereafter, approaching full recovery by day 17 onwards (Fig. 1A). Mechanical hypersensitivity of the hind paw (allodynia) was apparent alongside joint swelling, yet it persisted beyond the resolution of overt joint swelling, remaining highly significant relative to mice treated with control serum, at day 25 (Fig. 1B). All experiments herein were conducted using male mice. However, comparable observations in both clinical scores and mechanical hypersensitivity were seen in female mice treated with K/BxN serum (Supplemental Figure 1A, B, available at http://links.lww.com/PAIN/B11).

### 3.1. Monocytes/macrophages in the dorsal root ganglia

In the K/BxN model of inflammatory arthritis, the infiltration of nonclassical Ly6C^−^ monocytes into the joints is crucial for the initiation, progression, and resolution of joint swelling.^17^ As pain is a cardinal sign of inflammation, joint monocytes/macrophages are likely to contribute to nociception by releasing mediators that sensitise the peripheral terminals of nociceptive fibres. In this study, with the aim to assess whether immune cell infiltration occurred distant from the site of arthritic joint swelling, we examined the lumbar DRG, which is the site where the cell bodies of sensory neurons innervating the ankle joints are located (Figs. 1C–G). We selected 2 time points after serum transfer as follows: day 5 when swelling and mechanical hypersensitivity are both present and day 25 when mechanical hypersensitivity is still significantly maintained, but swelling has subsided. We observed that at day 5, F4/80^+^ cells (monocytes/macrophages) were present in DRG of control serum-treated group (Figs. 1C and G), and cell numbers were not different in DRG of the K/BxN serum-treated group (Figs. 1D and G). However, at day 25, the number of monocytes/macrophages was higher in DRG of K/BxN-treated than in control serum-treated mice (Figs. 1E–G).

These data suggest that monocytes/macrophages invade the lumbar DRG, and significant infiltration occurs in association with pain when joint swelling subsides (day 25).

### 3.2. Lipid mediator profiling in dorsal root ganglia

**Figure 2.**
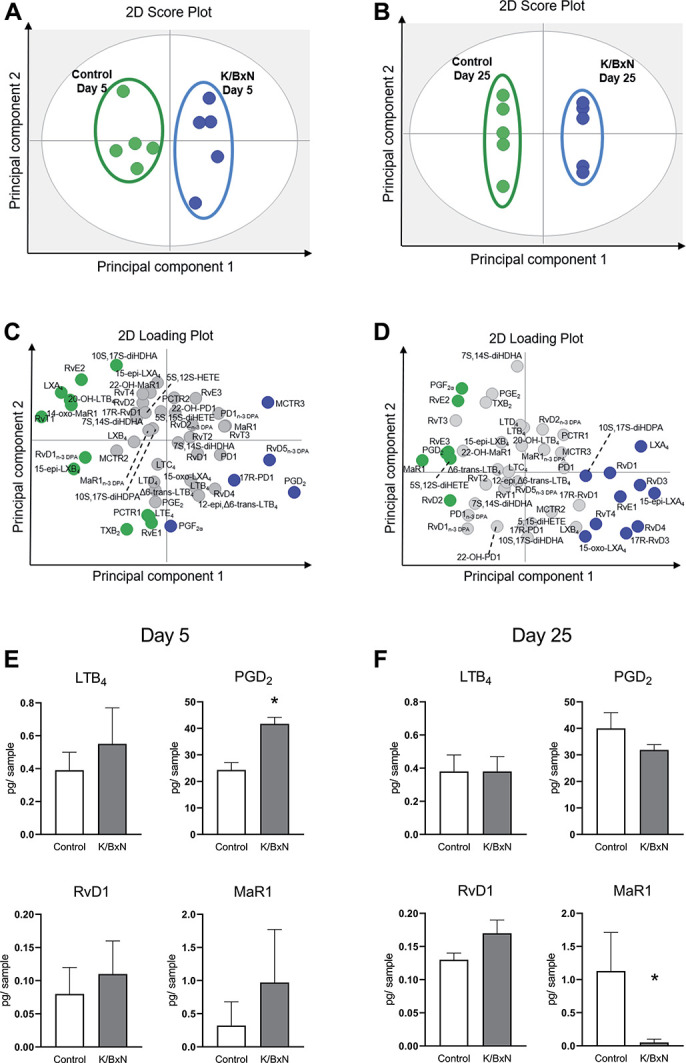
Distinct lipid mediator profiles in lumbar DRG at day 5 and day 25 after K/BxN serum transfer. Orthogonal partial least square to latent structure-discriminant analysis was used to generate 2 principal component functions from bioactive lipid mediator levels. (A and B) 2D score plot, gray ellipse denotes 95% confidence regions and (C and D) corresponding loading plots, LM coloured displayed a variable importance in projection coefficient ≥1. (A and C) 5 days or (B and D) 25 days after K/BxN serum transfer. (E and F) Quantification of selected lipid mediator levels at 5 and 25 days after K/BxN serum transfer. *P<0.05, Unpaired Student *t* test. Data are mean ± SEM; n = 5 mice per group. DRG, dorsal root ganglia; LM, lipid mediator.

To further our knowledge of mechanisms that could be potentially altered within the DRG at days 5 and 25 after serum transfer, we conducted LC-MS/MS–based LM profiling of arachidonic, eicosapentaenoic, docosahexaenoic, and docosapentaenoic acid bioactive metabolomes (>100 molecules) (Supplemental Figure 2A, available at http://links.lww.com/PAIN/B11). Our rationale was to link our data with known pronociceptive LMs as well as with the more recently described proresolving LMs, which carry antinociceptive actions^23,30^ (Supplemental Figure 2B, available at http://links.lww.com/PAIN/B11). At both day 5 and day 25, the measurement of over 100 essential fatty acid–derived molecules in DRG extracts, including mediators, pathway markers, and further metabolites of the omega-3 and omega-6 bioactive metabolomes, revealed striking differences between control and K/BxN DRG, as indicated by principal component analysis (Supplemental Table 2, available at http://links.lww.com/PAIN/B11; and Figs. 2A–D). At day 5, dwelling into the specific molecules allowed us to identify lipoxins and resolvins as abundant in control samples, whereas prostaglandins characterised DRG from mice challenged with K/BxN serum. In this article, PGD_2_ was the most significant discriminator (PGD_2_—control 24.3 ± 2.8; K/BxN: 41.76 ± 2.5 pg/sample), while leukotriene B_4_, resolvin D1, and maresin 1 (MaR1) levels were not altered (Fig. 2E). Intriguingly, at day 25, several proresolving mediators could be identified in control DRG samples, which were not present in the K/BxN-treated samples. Specifically, we observed a significant decrease in the proresolution and antinociceptive LM MaR1 levels vs control (nonarthritic) DRG (Supplemental Table 2, available at http://links.lww.com/PAIN/B11; and Fig. 2F). These data indicate a potential functional connection between decreased MaR1 levels in DRG and persistent allodynia dissociated from joint pain.

### 3.3. MaR1 application inhibits nociceptive neurons activation

**Figure 3.**
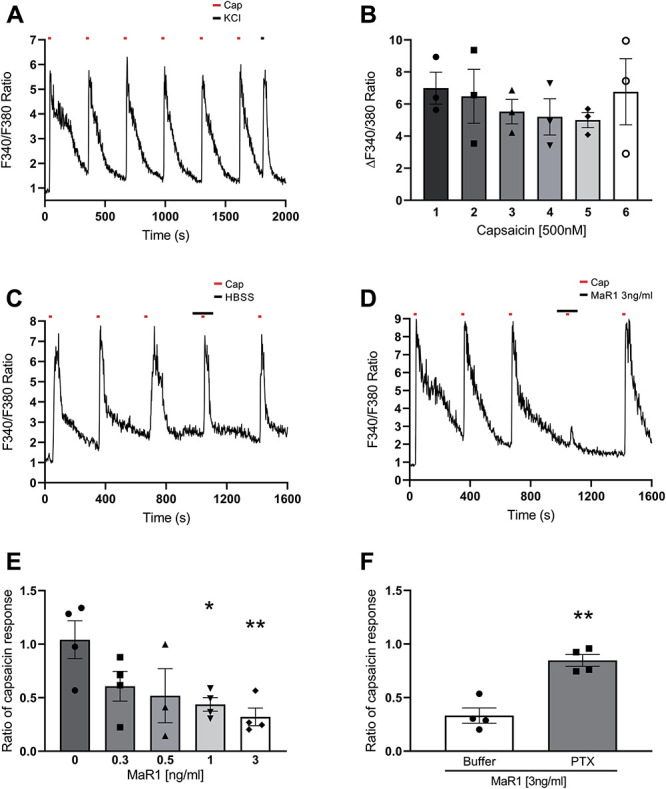
MaR1 reduces capsaicin-induced increase of intracellular Ca^2+^ in cultured DRG neurons through GPCRi/o pathway-mediated mechanisms. (A) Calcium imaging trace of neurons loaded with fura-2-AM (3 µM, 30 minutes) and then exposed to 6 capsaicin pulses (500 nM, 15 seconds/pulse) (red dots) at 5-minute intervals, before final application of KCl (50 mM, 15 seconds) (black dot). (B) Quantification of calcium responses after 6 consecutive applications of capsaicin. Data are mean ± SEM; n = 3 experiments. (C and D) Representative calcium traces of neurons exposed to HBSS buffer control (90 seconds) or MaR1 (3 ng/mL, 90 seconds) with the fourth capsaicin pulse. (E) Capsaicin response ratio (response 4/3) in the presence of MaR1 (0.83-8.3 nM for 90 seconds) during fourth capsaicin pulse. Data are mean ± SEM; n = 4 experiments. (F) Capsaicin response ratio in the presence of MaR1 (8.3 nM for 90 seconds) during fourth capsaicin pulse in neurons that were preincubated in pertussis toxin (GPCRi/o inhibitor) (500 ng/mL, 18 hours). *P < 0.05, **P < 0.01, one-way ANOVA, post hoc Tukey. Data are expressed as mean ± SEM; n = 4 experiments. ANOVA, analysis of variance; DRG, dorsal root ganglia.

MaR1 is a potent macrophage-derived proresolution LM,^23^ whose actions may not be limited to a specific cell type. Dorsal root ganglion tissue contains a mixed cell population of which both neurons and immune cells are present, with active cellular crosstalk during pain states.^26^ Thus, we investigated the possible effects of exogenous MaR1 on primary neurons and macrophages. In cultured DRG neurons, we assessed responses to capsaicin (TRPV1 agonist) using calcium imaging and CGRP release. In calcium imaging experiments, because of the fact that capsaicin application induces desensitisation of the TRPV1 channel, we first established that consecutive application of capsaicin (500 nM for 15 seconds applied at 5 minutes intervals) resulted in consistent increase in intracellular calcium concentration in cells that subsequently responded to KCl additions (Figs. 3A and B). We observed the smallest change in neuronal calcium influx between the third and fourth capsaicin pulses (Fig. 3B). Therefore, we applied increasing concentrations of MaR1 (0.3- to 3-ng/mL) both before and during the fourth capsaicin pulse. The addition of MaR1 caused a significant reduction of intracellular calcium ions at 1- and 3-ng/mL MaR1 concentrations (Figs. 3C–E). Evidence suggests MaR1 may exert inhibitory effects on TRPV1 currents through engagement of GPCR pathways.^23^ Consistently, we found that after pretreatment with the Gi/o-coupled GPCR blocker PTX (500 ng/mL), MaR1 no longer reduced capsaicin effects in cultured DRG neurons (Fig. 3F).

When measuring capsaicin-induced CGRP release from DRG neurons in culture, treatment with MaR1 at 100 nM, but not 1 and 10 nM, significantly reduced the release of CGRP by approximately 60%. Gabapentin, which was used as positive control, produced comparable inhibition at 1 nM and approximately 85% inhibition of release at 10 nM (Supplemental Figure 4D, available at http://links.lww.com/PAIN/B11).

In summary, we confirmed that MaR1 attenuates capsaicin-induced responses of nociceptive DRG neurons, a mechanism abolished by PTX pretreatment. However, critically, the effects of MaR1 were less considerable than those exerted by gabapentin.

### 3.4. Macrophage proinflammatory cytokine signalling is attenuated by MaR1 application

**Figure 4.**
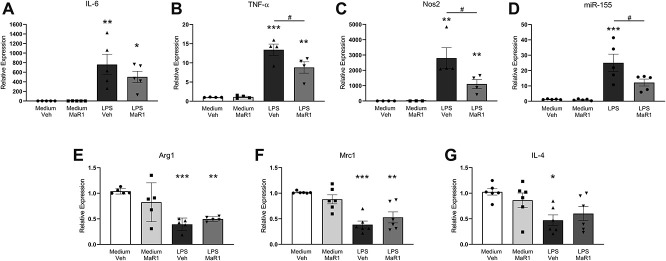
MaR1 reduces expression of proinflammatory markers in macrophages challenged with lipopolysaccharide. Cultured peritoneal macrophages incubated with or without LPS (100 ng/mL for 3 hours), followed by MaR1 (3 ng/mL = 8.3 nM) or vehicle for 5 hours. Quantification of mRNA for (A-D) proinflammatory markers and (E-G) anti-inflammatory markers assessed by qPCR. *P < 0.05, **P < 0.01, ***P < 0.001 compared with medium/vehicle controls; #*P* < 0.05, one-way ANOVA, post hoc Tukey. Data are mean ± SEM; n = 4 to 6 experiments. ANOVA, analysis of variance; LPS, lipopolysaccharide; qPCR, quantitative polymerase chain reaction.

In addition to modulating nociceptive-like activation of neurons, evidence suggests that MaR1 can influence macrophage phenotype, polarising bone-derived macrophages towards an M2 proresolution phenotype.^8^ Thus, we investigated the effect of MaR1 on cytokine expression and expression of miR-155 in peritoneal macrophages challenged with LPS. As expected, LPS incubation led to a significant increase in mRNA expression of proinflammatory cytokines IL-6, TNF-α, as well as of Nos2 and upregulated miR-155 (Figs. 4A–D) together with a downregulation of anti-inflammatory markers Arg1, Mrc1, and IL-4 (Figs. 4E–G). MaR1 (3 ng/mL) application 5 hours after LPS led to a significant reduction in TNF-α levels, Nos2, and miR-155 expression, as compared to vehicle-treated controls (Figs. 4A–D). MaR1 application after LPS exposure, however, did not significantly alter expression levels of the proresolution markers assessed (Figs. 4E–G). Similarly, MaR1 application alone in the absence of LPS had no effect on expression of either proinflammatory or anti-inflammatory markers (Figs. 4A–G).

These findings suggest that MaR1 reduces proinflammatory phenotype in macrophages after inflammatory insult with no effect on unchallenged macrophages.

### 3.5. MaR1 administration attenuates mechanical hypersensitivity

Our lipidomic analysis showed that MaR1 levels were significantly lower in K/BxN compared with control DRG at day 25 after serum transfer. Based on these observations and to provide in vivo relevance to our in vitro findings demonstrating MaR1 antinociceptive and anti-inflammatory actions on neurons and macrophages, we tested the effect of MaR1 administration on K/BxN mechanical hypersensitivity. Furthermore, we tested the effect of gabapentin, which has been previously shown to reverse K/BxN allodynia^20^ and acted as positive control (Supplementary Figure 4A–C, available at http://links.lww.com/PAIN/B11).

**Figure 5.**
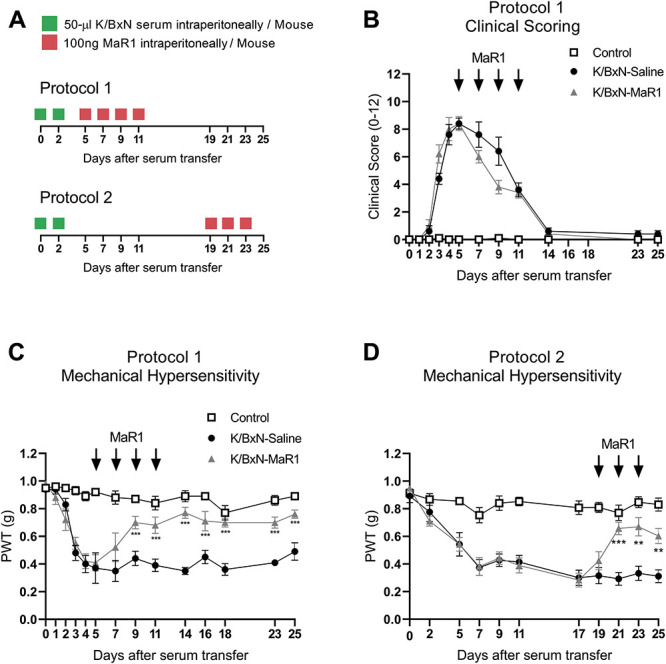
Repeated systemic administration of MaR1 results in sustained reversal of K/BxN serum-transfer–associated allodynia. (A) Timeline of experiments in which MaR1 (100 ng/mouse i.p) was repeatedly administered either at days 5, 7, 9, and 11 (protocol 1) or days 19, 21, and 23 (protocol 2) after serum transfer. (B) Clinical scoring of hind paws following MaR1 or saline administration. Control mice received control serum. (C) Reversal of mechanical hypersensitivity following third and fourth doses of MaR1 on days 9 and 11 after serum transfer. (D) Reversal of mechanical hypersensitivity after second and third doses of MaR1 on days 21 and 23 after serum transfer. Arrows indicate treatment days. **P < 0.01, ***P < 0.001 vs same day K/BxN-Saline group (closed circles), two-way ANOVA, post hoc Tukey. Data are mean ± SEM; n = 8 animals per group. ANOVA, analysis of variance.

MaR1 was administered every other day either at peak joint swelling and allodynia (from day 5 to day 11) (protocol 1) or from day 19 to day 23 (protocol 2) after serum transfer when allodynia persists while joint swelling subsides (Fig. 5A). Acute systemic injection of MaR1 (100 ng/mouse, intraperitoneally) on day 5 altered neither joint swelling nor mechanical hypersensitivity as assessed 1 hour after injection (Figs. 5B and C). Afterwards, while joint swelling was not significantly affected by MaR1-repeated administration (Fig. 5B), a second injection of MaR1 resulted in attenuation of mechanical hypersensitivity, which was significantly reversed after the third and fourth injections (Fig. 5C). The antinociceptive effect of MaR1 was maintained until day 25, which was 14 days after the last administration (Fig. 5C). A remarkable long-lasting antinociceptive action was also observed when female K/BxN serum-treated mice were treated on days 5, 7, 9, and 11 (protocol 1) (Supplemental Figure 1A and B, available at http://links.lww.com/PAIN/B11). MaR1 treatment administered in the absence of overt joint swelling on days 19, 21, and 23 (protocol 2) (Fig. 5D) also showed antinociceptive effects. Yet again, MaR1 acute administration was ineffective while second and third doses reverted mechanical hypersensitivity (Fig. 5D). As expected, gabapentin reverted allodynia 1 hour after the first administration to K/BxN mice on day 19 and maintained efficacy after the second and third doses on days 20 and 21. The effect of gabapentin has washed out 24 hours after the last dose (Supplemental Figure 4C, available at http://links.lww.com/PAIN/B11).

Together, these results highlight the lack of MaR1 acute efficacy, and the incurrence of a long-lasting antinociceptive effect of MaR1 in inflammatory arthritis, when administered systemically either in the presence or absence of joint swelling. As MaR1 is a proresolving mediator that derives from macrophages and has potent anti-inflammatory actions,^23^ we assessed whether monocyte/macrophage infiltration in DRG was altered in concomitance to the antinociceptive effect.

### 3.6. MaR1 treatment is associated with reduced monocyte/macrophage infiltration in the dorsal root ganglia

**Figure 6.**
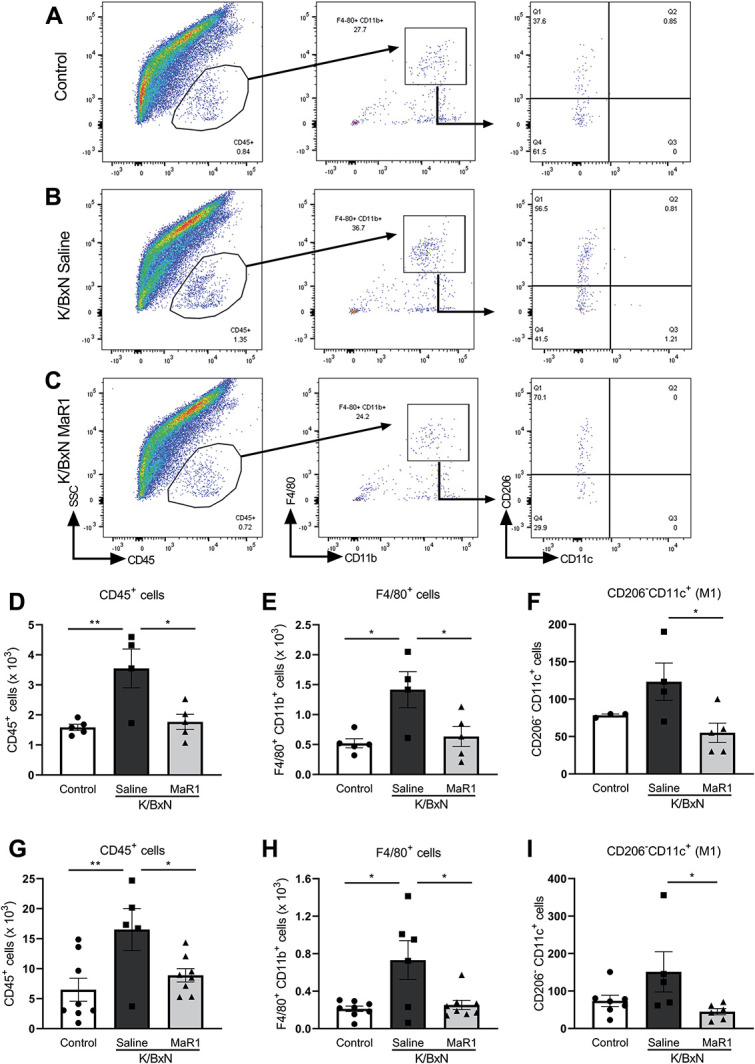
MaR1 treatment is associated with a decrease of immune cells and M1 macrophages recruitment in DRG after K/BxN serum transfer. (A–C) Representative scatterplots of immune cells sorted from lumbar and cervical DRG dissected at day 25 after transfer of control serum, K/BxN serum or K/BxN serum and MaR1 (4 doses, days 5-11 after K/BxN serum transfer, protocol 1). Cells were gated on CD45^+^, F4/80^+^, and CD11b^+^. Macrophages were defined as CD45^+^F4/80^+^CD11b^+^ and further analysed for M1 (CD11c^+^ CD206^−^) and M2 (CD11c^−^CD206^+^). (D–F) Bar charts representing numbers of leukocytes (CD45^+^ cells), macrophages (CD11b^+^F4/80^+^) and M1 macrophages (CD206^−^CD11c^+^) 14 days after last MaR1 dose (protocol 1); *P < 0.05, **P < 0.01, one-way ANOVA, post hoc Tukey. Data are mean ± SEM; n = 3 to 5 animals per group. (G–I) Bar charts representing numbers of leukocytes (CD45^+^ cells), macrophages (CD11b^+^F4/80^+^), and M1 macrophages (CD206^−^CD11c^+^) 2 days after last MaR1 dose (protocol 2); *P < 0.05, **P < 0.01, one-way ANOVA, post hoc Tukey. Data are mean ± SEM; n = 6 to 8 animals per group. ANOVA, analysis of variance; DRG, dorsal root ganglia.

Monocyte/macrophage infiltration in DRG contributes to neuronal sensitisation through the release of proinflammatory mediators.^22,26^ Thus, we evaluated whether MaR1 treatment affected this phenomenon and performed flow cytometry analysis of leukocytes, which infiltrated in the DRG on day 25 (gating strategy in Figs. 6A–C), which was either 14 (protocol 1) or 2 days (protocol 2) after the end of MaR1 treatments in K/BxN inflammatory arthritis. We observed that saline-treated K/BxN serum-transfer DRG contained a higher number of leukocytes (CD45^+^ cells) (Figs. 6A, B, and D) and macrophages (F4/80^+^CD11b^+^ cells) compared with saline-treated control serum DRG (Figs. 6A, B, and E); these cells were proinflammatory macrophages (M1; CD206^−^ CD11c^+^) (Figs. 6A, B, and F). At 2 weeks after the fourth dose, MaR1-treated K/BxN serum-transfer DRG contained significantly less leukocytes (CD45^+^) and macrophages (F4/80^+^CD11b^+^ cells) compared with saline-treated K/BxN (Figs. 6B–E). Importantly, the number of M1 macrophages (CD206^−^ CD11c^+^) was also significantly reduced (Figs. 6B, C, and F). Leukocyte analysis in DRG obtained 2 days after the last dose of MaR1 (protocol 2) also revealed that MaR1-treated K/BxN serum-transfer DRG contained less leukocytes (CD45^+^ cells), macrophages (F4/80^+^CD11b^+^ cells), and M1 macrophages (CD206^−^ CD11c^+^) compared with saline-treated K/BxN (Figs. 6G–I). Moreover, when examining leukocytes in the paws, in agreement with Misharin et al. (2016), we confirmed the presence of M2 macrophages in larger numbers than M1 macrophages (Supplemental Figure 3A–F, available at http://links.lww.com/PAIN/B11) and observed that MaR1-treated K/BxN serum-transfer paws contained significantly lower numbers of M1 cells (Supplemental Figure 3A–C and E, available at http://links.lww.com/PAIN/B11).

Altogether, these observations suggest that MaR1 treatment reverses K/BxN serum-transfer allodynia and reduced the number of proinflammatory macrophages in the DRG.

## 4. Discussion

In the K/BxN model of inflammatory arthritis 25 days after serum transfer, which mimics pain dissociated from joint inflammation in people with RA, we show that in lumbar DRG, where cell bodies of sensory neurons are located distal to the site of overt swelling in the joints, monocytes/macrophages are present in large numbers. Furthermore, still in DRG, while the expression of proinflammatory LMs is unaltered, the expression of proresolving LMs is decreased in concomitance to persistent allodynia dissociated from joint swelling. Thus, we provide evidence for an imbalance in LM levels between K/BxN and control DRG in association with allodynia that persists after resolution of joint swelling. We specifically observed reduced levels of the proresolution LM MaR1 at time points where allodynia occurs in the absence of overt joint swelling, yet macrophage infiltration arises in lumbar DRG. Consistent with the reduction in MaR1 expression as being correlated with allodynia, repeated systemic administration of MaR1 led to reversal of mechanical hypersensitivity by the third dose, and this effect was maintained for up to 2 weeks, a time point at which leukocyte and macrophage numbers in MaR1 treatment DRG were significantly lower than in vehicle treatment DRG. These observations suggest that a brief repeated-dosing protocol of MaR1 administration affects K/BxN serum-transfer–associated allodynia and the number of macrophages in the DRG.

In experimental inflammation, an important role has been identified for proresolving LMs, which control the second phase of the inflammatory response ensuring its timely and spatial restriction.^24^ Thus, these autacoids from the omega-3 and omega-6 metabolomes have been structurally elucidated and described to counteract the actions of proinflammatory LMs such as prostaglandins and leukotrienes. Malfunction or inadequate biosynthesis of proresolving mediators can contribute to the persistency of signs of inflammation of which nociception is one of the most relevant in the context of arthritis. The levels of proinflammatory LMs are increased in inflamed K/BxN paws.^19^ Here, we demonstrate in the same model changes to bioactive LMs in DRG, distant from the paws: using LC-MS/MS–based LM profiling, we identified the presence in DRG of LMs, both proinflammatory and proresolving ones. In the presence of overt joint swelling and allodynia on day 5 after serum transfer, we observed increased DRG levels of PGD_2_, which is pronociceptive.^28^ However, by day 25, where allodynia is present in the absence of joint swelling, PGD_2_ levels were no longer heightened in K/BxN DRG, and we observed lower levels of MaR1, which is a proresolution LM.^23^ Therefore, our analysis indicates distinct lipid profiles in DRG at day 5 (peak joint swelling) and day 25 (postjoint swelling) demonstrating that alternate mechanisms may be in place further away from the joint leading to maintained pain in RA dissociated from joint swelling.

MaR1 was identified as a macrophage-derived proresolving LM, which exerts potent modulatory actions both on immune cells and neurones.^23^ When added to macrophages, MaR1 modulates their functions promoting phagocytosis and efferocytosis, as well as favouring an M2 polarisation, all effects mediated by the high affinity to leucine-rich repeat containing G protein–coupled receptor 6 (LGR6).^3^ In the context of nociception and neuronal activity, MaR1 displays acute antinociceptive action in models of neuropathic and inflammatory pain after systemic and intrathecal injections.^9,23^ In DRG neurons, MaR1 inhibits TRPV1 receptor-mediated currents and reduces the capsaicin-induced release of CGRP.^9,23^ We have confirmed in vitro that MaR1 inhibits sensory neuron activity, reducing both capsaicin-induced calcium entry and CGRP release from cultured DRG, in a dose-dependent manner. Interestingly, although we observed MaR1-mediated reductions in capsaicin-induced CGRP release from sensory neurons, far greater MaR1 doses were required in comparison with gabapentin. In vitro differences in potency between gabapentin and MaR1 with respect to neuronally targeted actions are mirrored in the K/BxN serum-transfer model where, ourselves and others, have observed an antihyperalgesic ability of gabapentin within hours of systemic administration.^20^ Whereas, we observed no acute reversal of K/BxN-associated mechanical hypersensitivity after the first administration of MaR1. Previous studies show that when administered using intrathecal injection, MaR1 displays antihyperalgesic effects within hours in models of inflammatory pain and perioperative pain,^20,31^ possibly due to higher MaR1 concentrations locally in the spinal cord as a result of the administration route. Together, this evidence suggests that while MaR1 can reduce nociceptive responses by DRG neurons in culture, MaR1 treatment in vivo requires MaR1 local levels in the spinal cord level that are higher than those achieved after systemic treatment to observe the rapid antiallodynic effects, probably mediated at neuronal level.

In the K/BxN serum-transfer model, we believe, after systemic MaR1 administration, it is unlikely that a TRPV1-mediated mechanism in neurons plays a significant role in MaR1 antinociceptive effect in K/BxN-associated allodynia in arthritic settings. Instead, we suggest that MaR1 exerts an autocrine function in macrophages in DRG resulting in reduction of both proinflammatory macrophage recruitment and release of proinflammatory cytokines, which are known to sensitise sensory neurons and facilitate nociceptive signalling.^14^ Indeed, we provide in vivo evidence that repeated MaR1 treatment is associated with a decrease in M1 macrophages in DRG. However, the anti-inflammatory effects of MaR1 are not exclusive to the DRG. For instance, after systemic MaR1 treatment, fewer M1 macrophages were also observed in the arthritic hind paw. Therefore, it is possible that, in addition to the DRG, modulation of M1 macrophages in the paw may contribute to the antinociceptive action of MaR1, after K/BxN serum transfer. In vitro evidence also highlights that MaR1 reduces LPS-induced expression of proinflammatory cytokines TNF-α and Nos-2 in cultured peritoneal macrophages. In agreement, previous studies using human-derived macrophages showed MaR1 to be involved in macrophage switching from M1 to M2 phenotype.^8^ Intriguingly, our data indicate that MaR1 may be reducing proinflammatory macrophage signalling through modulation of miR-155, a known driver of inflammatory M1 macrophage phenotype, which regulates TNF-α and Nos production and Nf-κB signalling.^11^ Previous studies have shown that MaR1 treatment reduces proinflammatory cytokine protein expression in the spinal cord, after neuropathic injury,^10^ In agreement, based on our findings from cultured peritoneal macrophages, we would expect repeated MaR1 treatment to also reduce proinflammatory cytokine production in DRG after K/BxN serum transfer.

Overall, although we cannot dismiss neuronal-mediated antinociceptive abilities of MaR1, our data suggest that the delayed antiallodynic effects of systemic MaR1 administration is due to this specialised proresolving mediator having a long-term effect on macrophages, which is predominant over the effects on neurons.

Neuroimmune interactions are known to play a role in both the initiation and maintenance of chronic pain states.^12^ In the K/BxN model of inflammatory arthritis, infiltration of nonclassical Ly6C^−^ monocytes into the joints is crucial for the initiation, progression, and resolution of joint swelling.^17^ Ly6C^−^ monocytes are recruited to synovium during the early phase of the model, where they display plasticity, changing from a macrophage M1 (proinflammatory) to a majority of the M2 (anti-inflammatory) phenotype in situ.

We observed macrophage infiltration to occur in DRG, when pain is present in the absence of peripheral joint swelling. Therefore, infiltrating macrophages may be involved in pain maintenance in this model. Dorsal root ganglia macrophage infiltration and proliferation are known to occur in models of neuropathic pain^21,25,26^ and inflammatory pain.^22^ In the K/BxN serum-transfer model, monocytes/macrophages in the DRG are likely to have infiltrated through the endothelium in response to sensory neuron stress, triggered by peripheral inflammation.^13^ Indeed, in experimental arthritis, endothelial upregulation of vascular cell adhesion molecule 1 (VCAM-1) in DRG is associated with monocytes-macrophage infiltration, which is correlated with allodynia ipsilateral to the inflamed joints.^13^ Moreover, macrophages are activated and can subsequently sensitise nociceptive neurons, downstream of TNF-α–mediated activation.^22^ We speculate that DRG macrophage infiltration in the K/BxN serum-transfer model may occur through similar mechanisms whereby endothelial cell activation and upregulation of vascular cell adhesion molecule-1 (VCAM-1) can occur allowing macrophages to migrate through the vessel walls into the DRG tissue.

In conclusion, in inflammatory arthritis, the behavioural allodynia that persists even after resolution of joint swelling is associated with the presence of proinflammatory macrophages in DRG, which produce lower levels of the proresolution LM MaR1. Supplementation of MaR1 exerts significant and long-lasting antihyperalgesic actions and reduces the number of proinflammatory macrophages in the DRG. Therefore, we suggest that proresolution rather than anti-inflammatory strategies open innovative avenues for the treatment of RA pain that persists when joint inflammation enters remission after biologic or nonbiologic disease-modifying antirheumatic drug treatment.

## Conflict of interest statement

The authors have no conflicts of interest to declare.

## Appendix A. Supplemental digital content

Supplemental digital content associated with this article can be found online at http://links.lww.com/PAIN/B11.

## Supplementary Material

SUPPLEMENTARY MATERIAL
