# Supplementary material for: Imbalance of proresolving lipid mediators in persistent allodynia dissociated from signs of clinical arthritis
Source: Pain. 2020 May 4;161(9):2155–66. doi: 10.1097/j.pain.0000000000001908 (PMC7431142; doi:10.1097/j.pain.0000000000001908)
Supplement: SUPPLEMENTARY MATERIAL [file jop-161-2155-s001.pdf]

SUPPLEMENTAL DATA

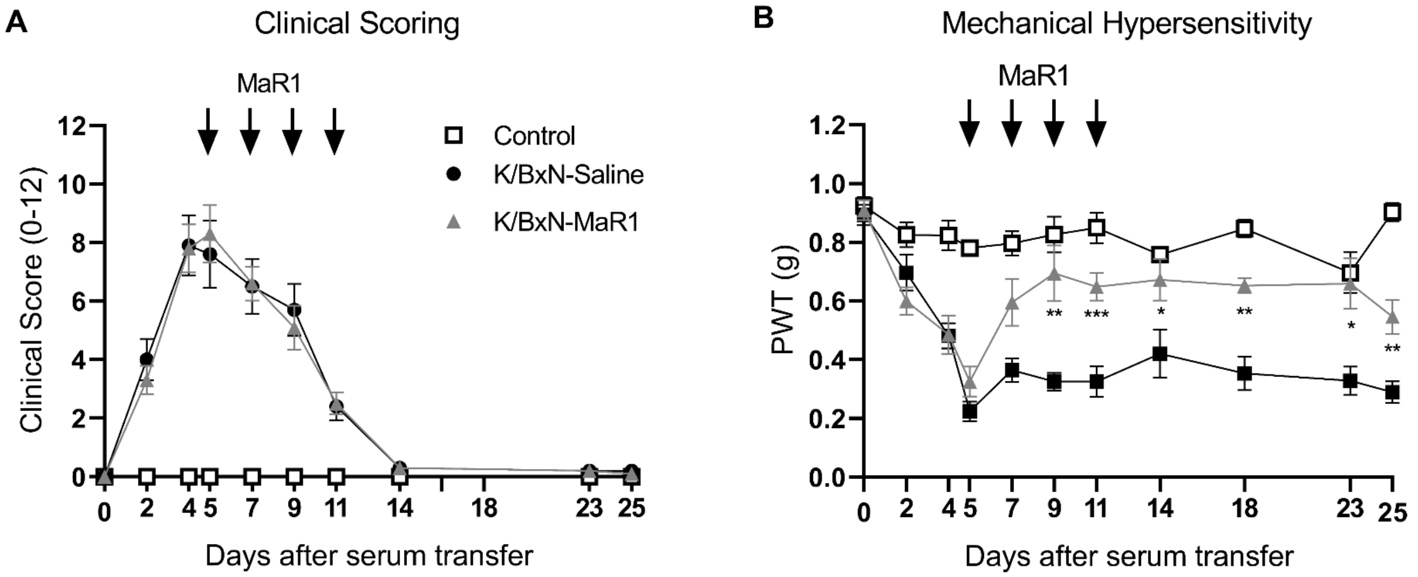

Supplemental Figure 1. Female K/BxN serum treated mice display mechanical hypersensitivity persisting beyond resolution of joint swelling, with recovery from allodynia following repeated MaR1 treatment (A) Clinical signs of arthritis following K/BxN serum transfer (2 x 50 µl intraperitoneal injections on days 0 and 2) evaluated using a 12-point clinical arthritis scoring of mouse paws (B) Mechanical hypersensitivity assessed using von Frey filaments following K/BxN serum transfer. Arrows indicate treatment days where 100ng MaR1 or Saline was administered i.p. ***p<0.001, **p<0.01 or *p<0.05, versus same day control, Two-Way RM ANOVA, post-hoc Tukey. Data are expressed as mean ± SEM; n=10 female mice per group.

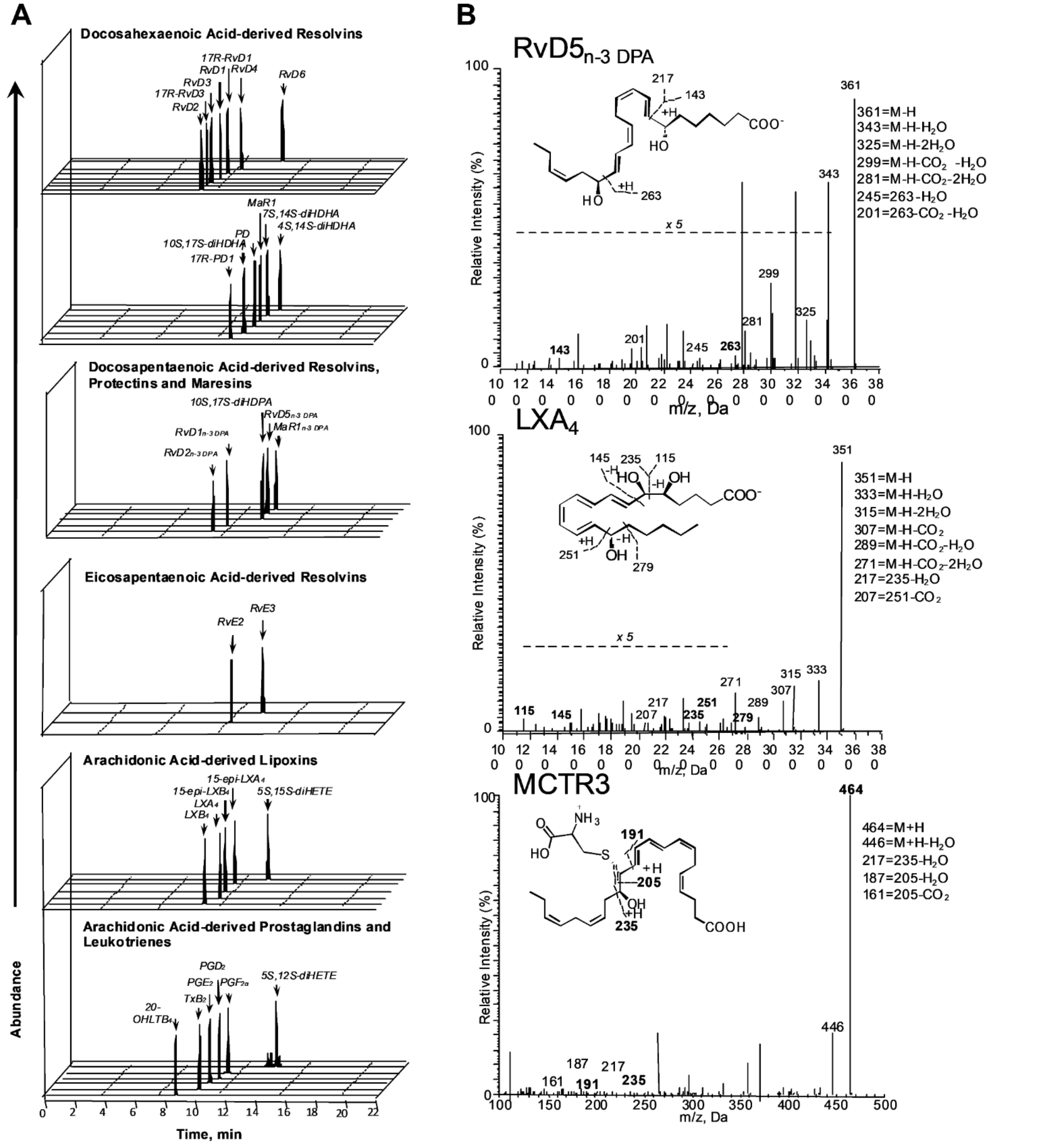

Supplemental Figure 2. Identification of bioactive lipid mediators in lumbar DRG after K/BxN serum transfer. Following solid phase extraction, lipid mediator levels were investigated using liquid chromatography tandem mass spectrometry (LC-MS/MS). (A) Representative multiple reaction monitoring (MRM) chromatograms from 10 mice for each of the identified lipid mediators in DRG. (B) MS-MS spectra employed for the identification of RvD5, LXA_4 and MCTR3.

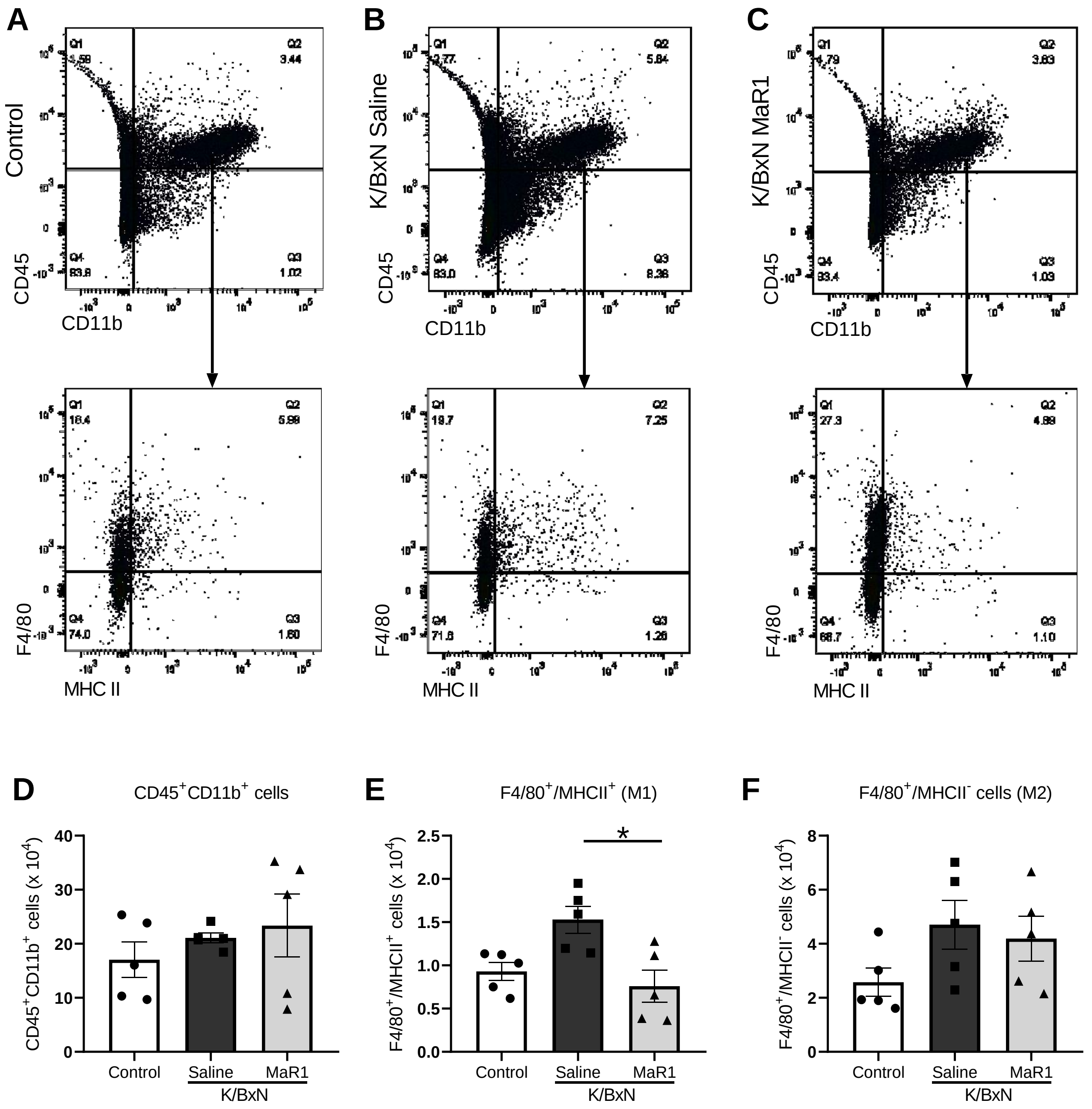

Supplemental Figure 3. Inflammatory M1 macrophage paw infiltration following K/BxN induction is attenuated by MaR1 treatment with no effect on majority M2 macrophages in the paw. (A-C) Representative scatterplots of immune cells sorted from fore paws and hind paws dissected at day 25 after transfer of control serum, K/BxN serum or K/BxN serum and MaR1 (3 doses, days 19-23 after K/BxN serum transfer, Protocol 2). Cells were gated on CD45+, CD11b+. Macrophages were defined as CD45+ CD11b+ F4/80+ and further analysed for M1 (F4/80+ MHCII+) and M2 (F4/80+ MHCII-) phenotypes. (D-F) Bar charts representing numbers of macrophages (CD45+CD11b+), M1 macrophages (F4/80+MHC+) and M2 macrophages (F4/80+MHC-) 2 days after last MaR1 dose (Protocol 2). * $p<0.05$ One-Way ANOVA, post-hoc Tukey. Data are mean ±SEM; *n*=5 animals per group.

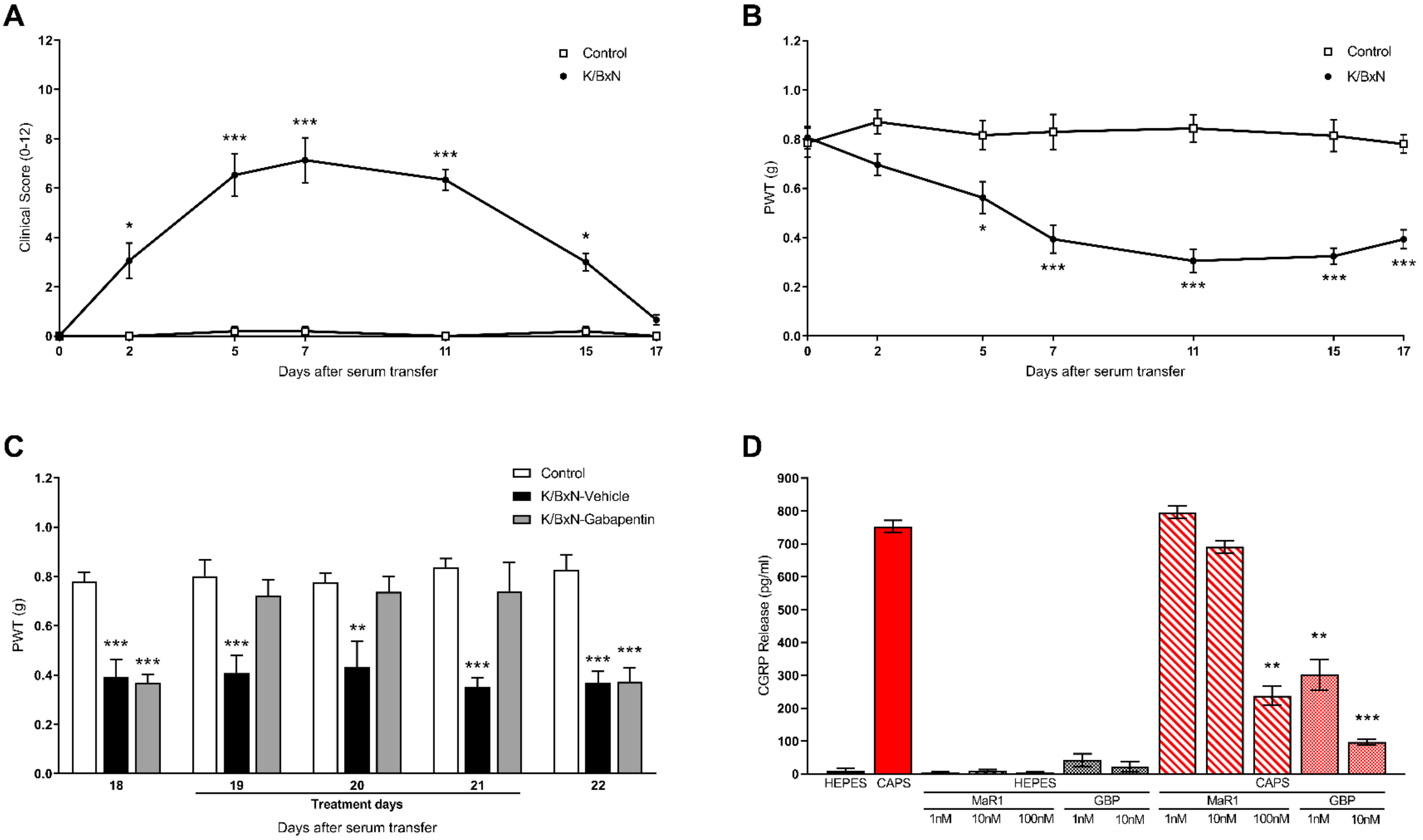

Supplemental Figure 4. Both MaR1 and gabapentin reduce capsaicin-induced CGRP release from cultured DRG neurons, with gabapentin also displaying rapid anti-allodynic effects in K/BxN serum transfer model when administered after cessation of joint swelling. (A) Assessment of mechanical hypersensitivity (hind paw withdrawal thresholds) and (B) clinical signs of arthritis following serum transfer, prior to gabapentin or vehicle treatment. Control mice received control serum. *** = p<0.001, ** = p<0.01, ** = p<0.05 compared to controls, Two-Way RM ANOVA, post-hoc Tukey. Data are expressed as mean ± SEM; *n*=15 animals per group (K/BxN), *n*=5 animals per group (Control). (C) Baseline mechanical thresholds recorded on day 18 after K/BxN serum transfer and after, at 1 hour after treatment with either gabapentin (60 mg/kg p.o.) or vehicle (dH$_2$0) on days 19, 20 and 21 after serum transfer, and 24 hours after the last gabapentin administration on day 22 . *** = p<0.001, ** = p<0.01 compared to controls, Two-Way RM ANOVA, post-hoc Tukey. Data are expressed as mean ±SEM; *n*=5 animals per group (D) Capsaicin-induced CGRP release in cultured DRG neurons in the absence or presence of MaR1 (1,10 or 100 nM) or gabapentin (1 or 10 nM). *** = p< 0.001, ** = p<0.01 compared to capsaicin treatment alone (solid red), one-way ANOVA, post-hoc Tukey. Data are expressed as mean ±SEM; *n*=3 cultures.

Target Gene	Forward Primer	Reverse Primer	Accession Number
Actb	5'- GGCTGTATTCCCCTCCATCG	5'- CCAGTTGGTAACAATGCCATGT	NM_007393.5
Arg1	5'- GTGAAGAACCCACGGTCTGT	5'- CTGGTTGTCAGGGGAGTGTT	NM_007482.3
Il4	5'- TGGAGCTGCAGAGACTCTTT	5'- CATGATGCTCTTTAGGCTTT	NM_021283.2
Il6	5'- GGCTGTATTCCCCTCCATCG	5'- CCAGTTGGTAACAATGCCATGT	NM_031168.2
Nos2	5'- GGCAAACCCAAGGTCTACGTT	5'- CTCAAGTTCAGCTTGGT	NM_010927.4
Mrc1	5'- CAGGTGTGGGCTCAGGTAGT	5'- TGTGGTGAGCTGAAAGGTGA	NM_008625.2
TNFa	5'- GTGCCTATGTCTCAGCCTCT	5'- TGGTTTGTGAGTGTGAGGGT	NM_013693.3
**ID-miRBase Version 19.0**	**Mature sequence 5'-3'**		**Accession number**
mmu-miR-155-5p	UUAAUGCUAAUUGUGAUAGGGGU		MIMAT0000165

Supplemental Table 1. Sequences of miRNAs analysed and primers used for real-time PCR analysis in this study. Primers were provided by Sigma Aldrich. miRNA sequences were provided by Exiqon.

			Lipid mediator levels (pg/sample)			
	Q1	Q3	Control Day 5	K/BxN Day 5	Control Day 25	K/BxN Day 25
**DHA Metabolome**						
RvD1	375	233	0.08 ± 0.04	0.11 ± 0.05	0.13 ± 0.01	0.17 ± 0.02
RvD2	375	141	0.12 ± 0.04	0.08 ± 0.06	0.15 ± 0.09	0.03 ± 0.04
RvD3	375	147	0.11 ± 0.09	-	0.04 ± 0.04	0.42 ± 0.16 *#
RvD4	375	101	0.07 ± 0.03	0.1 ± 0.04	0.04 ± 0.03	0.43 ± 0.18 *#
RvD5	359	199	-	-	-	-
RvD6	359	101	0.02 ± 0.02	-	-	0.03 ± 0.03
17R-RvD1	375	233	0.04 ± 0.03	0.02 ± 0.02	0.02 ± 0.03	0.06 ± 0.03
17R-RvD3	375	147	-	0.03 ± 0.02 *	-	0.03 ± 0.01 *
PD1	359	153	0.02 ± 0.03	0.09 ± 0.08	0.01 ± 0.02	0.03 ± 0.02
10S, 17S-diHDHA	359	153	0.17 ± 0.02	0.11 ± 0.06	0.04 ± 0.02	0.05 ± 0.03
17R-PD1	359	153	0.01 ± 0.01	0.02 ± 0.01	0.01 ± 0.01	0.01 ± 0.01
22-OH-PD1	375	153	0.04 ± 0.03	0.05 ± 0.03	0.04 ± 0.02	0.03 ± 0.02
PCTR1	650	231	0.51 ± 0.37	0.51 ± 0.36	0.26 ± 0.29	0.54 ± 0.37
PCTR2	521	231	0.27 ± 0.19	0.3 ± 0.34	0.44 ± 0.49	0.13 ± 0.15
PCTR3	464	231	-	-	-	-
MaR1	359	221	0.32 ± 0.36	0.97 ± 0.8	1.13 ± 0.58	0.05 ± 0.05 *
MaR2	359	191	-	-	-	-
22-OH-MaR1	375	250	0.42 ± 0.16	0.37 ± 0.13	0.52 ± 0.24	0.22 ± 0.2
14-oxo-MaR1	357	249	0.38 ± 0.19	- *	-	0.07 ± 0.07
7S, 14S-diHDHA	359	221	0.82 ± 0.63	0.43 ± 0.48	0.46 ± 0.33	0.17 ± 0.19
4S, 14S-diHDHA	359	101	-	0.03 ± 0.02	0.05 ± 0.04	-
MCTR1	650	191	13.01 ± 5.04	15.55 ± 4.21	25.72 ± 2.80 #	16.18 ± 2.31 *
MCTR2	521	191	0.97 ± 0.77	-	0.85 ± 0.95	-
MCTR3	464	191	0.49 ± 0.16	0.41 ± 0.13	0.42 ± 0.15	0.5 ± 0.13
**n-3 DPA Metabolome**						
RvT1	377	239	4.47 ± 1.12	1.92 ± 0.23 *	2.27 ± 0.83 #	2.01 ± 0.23 *
RvT2	377	197	0.06 ± 0.02	0.06 ± 0.02	0.08 ± 0.03	0.05 ± 0.03
RvT3	377	197	0.01 ± 0.01	0.02 ± 0.01	0.02 ± 0.01	0.01 ± 0.01
RvT4	361	211	0.02 ± 0.01	0.01 ± 0.01	0.01 ± 0.01	0.03 ± 0.02 #
$RvD1_{n\text{-}3\ DPA}$	377	143	0.39 ± 0.11	0.27 ± 0.05	0.39 ± 0.05	0.33 ± 0.05
$RvD2_{n\text{-}3\ DPA}$	377	143	0.09 ± 0.05	0.08 ± 0.04	0.12 ± 0.05	0.15 ± 0.1
$RvD5_{n\text{-}3\ DPA}$	361	199	-	0.15 ± 0.08 *	0.12 ± 0.09 #	0.1 ± 0.12
$PD1_{n\text{-}3\ DPA}$	361	183	0.04 ± 0.04	0.07 ± 0.03	0.07 ± 0.03	0.03 ± 0.03
10S, 17S-diHDPA	361	183	0.09 ± 0.04	0.06 ± 0.07	0.03 ± 0.02 #	0.06 ± 0.03
$MaR1_{n\text{-}3\ DPA}$	361	249	0.12 ± 0.10	0.06 ± 0.05	0.06 ± 0.06	0.08 ± 0.05
7S, 14S-diHDPA	361	249	0.12 ± 0.04	0.14 ± 0.07	0.13 ± 0.04	0.12 ± 0.06
**EPA Metabolome**						
RvE1	349	161	0.11 ± 0.04	0.09 ± 0.02	0.14 ± 0.05	0.29 ± 0.07*#
RvE2	333	199	0.06 ± 0.02	0.03 ± 0.02	0.07 ± 0.02	0.05 ± 0.01
RvE3	333	201	0.06 ± 0.04	0.14 ± 0.16	0.12 ± 0.06	0.3 ± 0.04
**AA Metabolome**						
LXA_4	351	115	0.04 ± 0.01	0.01 ± 0.01 *	0.02 ± 0.01	0.06 ± 0.01 *#
LXB_4	351	221	0.97 ± 0.56	0.71 ± 0.36	0.35 ± 0.13	0.75 ± 0.49
5,15-diHETE	335	235	3.15 ± 0.51	2.95 ± 0.46	3 ± 0.23	4.71 ± 0.55 #
15-epi-LXA_4	351	115	0.17 ± 0.09	0.04 ± 0.01	0.12 ± 0.04	0.1 ± 0.04 #
15-epi-LXB_4	351	221	0.09 ± 0.07	0.2 ± 0.14	0.35 ± 0.2	0.55 ± 0.06 #
13,14-dihydro-15-oxo-LXA_4	351	115	0.02 ± 0.01	0.02 ± 0.01	0.02 ± 0.01	0.02 ± 0
15-oxo-LXA_4	349	115	-	-	-	-
LTB_4	335	195	0.39 ± 0.11	0.55 ± 0.22	0.38 ± 0.1	0.38 ± 0.09
5S,12S-diHETE	335	195	0.09 ± 0.03	0.08 ± 0.03	0.18 ± 0.1	0.06 ± 0.02
12-epi, Δ6-trans-LTB_4	335	195	0.19 ± 0.04	0.25 ± 0.09	0.2 ± 0.05	0.18 ± 0.04
Δ6-trans-LTB_4	335	195	0.20 ± 0.08	0.19 ± 0.08	0.21 ± 0.08	0.17 ± 0.06
20-OH-LTB_4	351	195	0.04 ± 0.03	0.01 ± 0	0.04 ± 0.02	0.04 ± 0.02
LTC_4	626	189	8.39 ± 2.62	8.73 ± 3.23	9.54 ± 1.91	8.88 ± 1.99
LTD_4	497	189	1.17 ± 0.40	1.14 ± 0.65	0.97 ± 0.27	0.95 ± 0.21
LTE_4	440	189	0.42 ± 0.22	0.33 ± 0.37	- #	-
PGD_2	351	189	24.31 ± 2.79	41.67 ± 2.51*	39.97 ± 5.97 #	31.84 ± 2.06 #
PGE_2	351	189	16.34 ± 2.11	17.21 ± 3.35	22.68 ± 3.65 #	20.6 ± 2.23
$PGF_{2\alpha}$	351	193	3.33 ± 0.43	3.64 ± 0.69	4.9 ± 0.74 #	3.95 ± 0.63
TxB_2	369	169	5.80 ± 0.86	5.32 ± 1.1	8.17 ± 1.09 #	7.2 ± 1.26

Supplemental Table 2. Lipid mediators from DHA, n-3 DPA, EPA and AA bioactive metabolome identified in lumbar dorsal root ganglia (DRG) from control and arthritic K/BxN mice on days 5 and 25 post-immunisation. Q1: M-H (parent ion) and Q3 (daughter ion): diagnostic ion in the MS-MS *p< 0.05 versus respective control treated mice, # p<0.05 versus respective Day 5 values, unpaired student’s t-test. – for lipid mediator levels are below detection limits (approximately 0.1 pg). Data are expressed as mean ± SEM, n = 5 mice per group and presented as pg/sample
